# Network-Based and Binless Frequency Analyses

**DOI:** 10.1371/journal.pone.0142108

**Published:** 2015-11-03

**Authors:** Sybil Derrible, Nasir Ahmad

**Affiliations:** Complex and Sustainable Urban Networks (CSUN) Laboratory, University of Illinois at Chicago, Chicago, IL, United States of America; Universidad Rey Juan Carlos, SPAIN

## Abstract

We introduce and develop a new network-based and binless methodology to perform frequency analyses and produce histograms. In contrast with traditional frequency analysis techniques that use fixed intervals to bin values, we place a range ±*ζ* around each individual value in a data set and count the number of values within that range, which allows us to compare every single value of a data set with one another. In essence, the methodology is identical to the construction of a network, where two values are connected if they lie within a given a range (±*ζ)*. The value with the highest degree (i.e., most connections) is therefore assimilated to the mode of the distribution. To select an optimal range, we look at the stability of the proportion of nodes in the largest cluster. The methodology is validated by sampling 12 typical distributions, and it is applied to a number of real-world data sets with both spatial and temporal components. The methodology can be applied to any data set and provides a robust means to uncover meaningful patterns and trends. A free python script and a tutorial are also made available to facilitate the application of the method.

## Introduction

In an era of pervasive computing and data-driven decision-making, large amounts of data are being generated and collected every day, and making sense of these data is not straightforward [1,2]. The amount of information generated is so important that traditional data analytics are often unable to provide helpful insights. In particular, the problem of detecting meaningful patterns from the noise created by erroneous data or by the presence of significant outliers [3] is often hard to deal with. While these issues are becoming more challenging with new data loads [4], they have existed since the beginning of statistics [5]. Detecting these patterns is critical, however, if we are to gain a better understanding of the underlying processes behind the phenomena captured in a data set [6]. A common problem for instance is the use of the arithmetic mean as a representative value of a population. Indeed, most distributions are not symmetrical and the mean is therefore most often skewed from the *mode* of the distribution, which is arguably more representative (see Section A in S1 File), albeit harder to calculate in large samples.

Moreover, these meaningful patterns can have various shapes and are sometimes observed in the form of familiar distributions (e.g., normal, lognormal, logistic distributions). While many techniques exist to fit data to these distributions (e.g., linear regression, maximum likelihood, machine learning), most require us to have an initial estimation of the form of the fit for our data. This estimation is most commonly acquired by performing frequency analyses and plotting histograms [7]. Typical histograms show the frequency (i.e., number of occurrence) of events within fixed intervals, called *bins*. The sizes of these bins are semi-arbitrary inputs and selecting appropriate sizes is highly non-trivial [8]. Many rules exist (e.g., Sturges, Rice, Freedman-Diaconis), and the most utilized may be Scott’s rule defined as: $\frac{3.49\sigma }{\sqrt[{3}]{n}}$, where *σ* is the standard deviation of the sample and *n* is the size of the population. Although Scott’s rule calculates an optimal bin size for random normally distributed data, real-world data is often not normal, and applying Scott’s rule can fail to capture relevant information. This is typically the case for distributions that show multi-modal features that get absorbed in one single bin for instance. Furthermore, many data sets contain outliers (i.e., erroneous or invalidated data) that if not removed can bias the calculation of the parameters, for parametric distributions, that are then directly used to fit the distribution (e.g., errors in calculating the mean and standard deviation for a normal distribution). To detect these outliers, several techniques exist and they tend to look at the difference of individual values with the mean or their eccentricity from the interquartile range [9].

Here, we create and develop a new binless methodology that compares all values or a sample of values of a distribution with one another. Mathematically, for a distribution {*x*_*1*_, …, *x*_*n*_}, we say that value *x*_*j*_ is ‘similar’ to value *x*_*i*_ if (*x*_*i*_ - *ζ*) ≤ *x*_*j*_ ≤ (*x*_*i*_ + *ζ*), for a given range ±*ζ*. We can then identify the value that is the most ‘similar’ to any other values, therefore representing the mode of the distribution. Although the two are conceptually different, the methodology is close to the notion of homophily [10] in network science [11], where a graph *G* with *V* vertices/nodes and *E* edges/links is created and two individuals are connected if they share similar properties (e.g., income, interests). The foundation of the methodology is closest to kernel density estimation (KDE) in machine learning (see Section B in S1 File) although the procedures rapidly differ, notably in how the range ±*ζ* is determined.

The general methodology is explained in the next section and Section C in S1 File contains a simple example. We then present the general mechanism, a validation of the methodology on 12 simulated distributions, an application to three real-world data sets, and an application to a time series data set. Moreover, a free python script and tutorial were made freely available for download on the authors’ website at http://csun.uic.edu/data.html and on the popular code sharing platform GitHub at https://github.com/csunlab/nbfreq.

## Materials and Methods

### General Principles

The main mechanism of the methodology is to compare values of a distribution with one another by measuring whether they are similar or not (i.e., whether they lie within a certain range of one another). In other words, while traditional histograms have fixed ranges, this methodology adapts the ranges around individual values. Computationally, the procedure can be relatively slow, akin to KDE. For relatively small datasets, all values can be compared with one another, but typical sampling techniques can be used for larger data sets [4] (i.e., running the technique on a fix proportion of data points drawn randomly from the dataset—illustrated below).

Although conceptually different, the proposed method is identical to the formation of a network. In network science, a graph *G* is composed of a set of nodes/vertices *V* and links/edges *E*; *G* = {*V*,*E*}. If we assume that each value in the distribution is a node, then two nodes are connected if their values are within a certain range of one another. A network is analytically represented by its adjacency matrix *A*_*ij*_ in which each cell takes the value of 1 if nodes *i* and *j* are connected and 0 otherwise; more formally in our case:

$$A_{ij}\;=\;{\{}_{0\;\;\text{otherwise}}^{1\text{ if }\left(x_{i}-\zeta \right)\leq x_{j}\leq \left(x_{i}+\zeta \right)}$$ (1)

**Fig 1:**
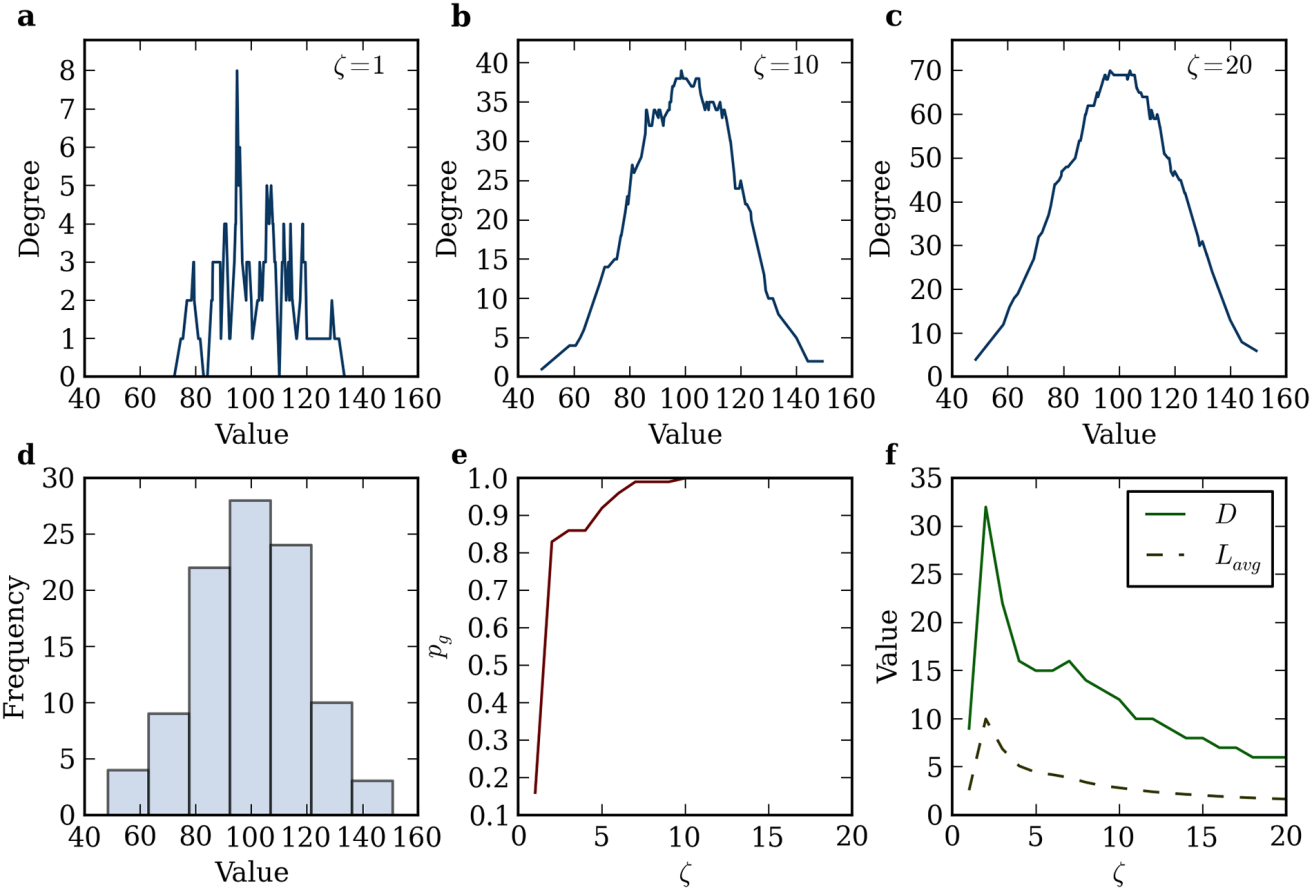
Impact of *ζ* on NB Methodology and Network Properties. (A)-(C) Scatter plots of values and their degrees (i.e., number of connections) for *ζ* = 1, 10, 20 respectively. (D) Histogram of distribution where the bin size of 14.70 was calculated using Scott’s rule. We can see that the right shape is obtained but the bins are large. (E) Evolution of *p*_*g*_ with *ζ*. (F) Evolution of the *D* and *L*_*avg*_ with *ζ*. In practice, we choose *ζ* as a low percentage of the median of the distribution, say 1%, and we then increase it gradually until the value of *p*_*g*_ becomes constant over several increases of *ζ* (this number of increases depends on the magnitude of increase between every *ζ*); here, we find the network becomes stable for *ζ* = 10.

Using this network analogy, the frequency/count in Fig 1 (detailed later) simply takes the form of the degree *d*_*i*_ of a node *i* (i.e., the number of connections), defined from *A* as:

$$d_{i}=\sum_{j=1}^{n}A_{ij}$$ (2)

A network-based histogram can therefore be constructed by plotting the degree of each node versus its value. The value of the node with the highest degree corresponds to the mode, *M*, of the distribution.

$$M=V\left(\text{max}\left(d_{i}\right)\right)$$ (3)

This is especially applicable for single mode distributions. A visual inspection of the distribution, by plotting the degree vs. value, is required to determine the values of other modes. Moreover, since we actually build the adjacency matrix *A* and along with this network analogy, a plethora of metrics can be used. Of relevance, the average shortest-path length, which is defined as the average of all shortest-path lengths, *L*_*avg*_, between every pair of nodes, becomes a measure of spread of a distribution akin to the standard deviation. The diameter of the network, *D*, which is the largest of all shortest-path lengths, gives us a different measure of spread.

### Outlier Detection

One feature of the methodology is its ability to detect outliers. Put simply, from Eq 1, we can see that not all values will necessarily be connected to other values if the value of *ζ* is too small to create a connection. Moreover, these outliers are not part of the largest cluster (discussed below for validation) and they can therefore be easily filtered out if desired. In other words, the values that are part of the largest cluster can be extracted for further analysis. Moreover, having low or nil degree values, outliers have no impact on the detection of the mode of the distribution as defined by Eq 3. In fact, we should note that the presence of outliers does not necessarily have a significant impact on many of the metrics calculated. For instance, path-lengths are calculated within clusters since a path between two unconnected nodes is infinite. The diameter of the network therefore takes the value of the diameter of the largest cluster.

### Runtime

As mentioned above and akin to KDE, the procedure can be relatively slow for large datasets. This is, however, generally the case for most techniques that involve the construction of a network. A shorter version of the entire code for the methodology was run on a MacBook Pro with a 2.6 GHz Intel Code i7 processor and 8 GB of memory. The code is in python, and processing time is therefore much slower than C or Java language, although we are making extensive use of NumPy [12]. Moreover, we are using the python igraph library [13] to detect the giant cluster; however, igraph calculates multiple metrics when ready an adjacency matrix, further slowing down the process. The code used for this section can be found in Section D in S1 File. It was run on seven data sets, ranging from 100 data points to 50,000 data points, randomly sampling a lognormal distribution ln*N*(1,0.5). The lognormal distribution with a large standard deviation is chosen because it has a fat right tail, thus exhibiting common behavior in real data sets. A total of 10 *ζ* coefficients (values ranging from 1% to 10% of the median) are tested for each samples. A simple bootstrapping technique with no replacement was used to sample some of the larger data sets. The bootstrapping results were satisfactory; i.e., the modes calculated with and without sampling were close and the shape of the distribution was the same. The runtime results are showed in Table 1; the first column displays the sample size, the second column displays the sampling coefficient when a bootstrapping method is applied, and the third column shows the runtime. The computer ran out of memory when processing 50,000 points. A caveat should be added, however. Runtime heavily depends on the data itself. More homogeneous data will create more links, which will slow down the process. Moreover, a pure C or C++ code would likely render much faster runtimes while handling more points.

**Table 1:** Runtime Evaluation.

Sample Size	Sampling	Runtime
100	-	0.0204 s
500	-	0.1720 s
1,000	-	0.6496 s
5,000	-	17.6039 s
10,000	-	1:20.74 min
20,000	20%	10.7596 s
20,000	50%	1:15.72 min
20,000	-	20:59.94 min
50,000	20%	1:15.21 min
50,000	50%	1:23:52 hr
50,000	-	out of memory

## Results and Discussion

### Validation

From Eq 1, the selection of *ζ* can have significant impacts on the results. A *ζ* too small may omit important connections, while a *ζ* too large artificially inflates the main mode of the distribution. The main objective is therefore to select a *ζ* for which the network properties have become stable. Because we are not fitting a distribution to the values, we cannot seek a method to minimize the mean squared error as is usually the case in statistics (see Section B in S1 File for KDE). Instead, we propose to look at the evolution of the number of nodes in the largest cluster. As we slowly increase the value of *ζ*, clusters (akin to communities) form and a larger / giant cluster rapidly emerges containing *V*_*g*_ nodes. We define the proportional number of vertices in the giant cluster as *p*_*g*_ = *V*_*g*_ / *V*. After a certain value of *ζ*, the giant cluster admits few or no new nodes, and *p*_*g*_ does not change, which suggests it has reached stability. An additional condition can be added to ensure a certain proportion of nodes are present in the giant cluster (e.g., *p*_*g*_ ≥ 0.68 akin to the number of values within one standard deviation for normal distributions). As an example, Fig 1(E) shows the increase of *p*_*g*_ with *ζ* for a 100 random normally distributed, *N*(100, 20), values.

Fig 1(A)–1(C) shows scatter plots of individual values vs. their degrees for three different *ζ*. We can see that a small *ζ* looks more like noise and it takes the shape of a normal distribution as we increase *ζ*, after which the entire distribution starts to inflate. In this particular case, *p*_*g*_ becomes stable at *ζ* = 10 (Fig 1(E)), where all nodes are part of the giant cluster, i.e., *p*_*g*_ = 1, which suggests there are no outliers in the distribution. Stability in *p*_*g*_ remains a user-defined notion at this point. For this work, we say that the network is optimal when *p*_*g*_ remains identical for several increases of *ζ*; the exact number of increases depends on the magnitude of each increase set. For large increases, fewer consecutive increases of *ζ* need to be identical. In the code provided on GitHub, we set this number to 30% of all the different values of *ζ* tested, but akin to most data mining techniques, several iterations with a visual inspection may be preferable.

One means to further validate visually *ζ* is to analyze the evolution of the diameter *D* and average path length *L*_*avg*_ of the network created. Fig 1(F) shows *D* and *L*_*avg*_ for the normal distribution generated. We can see that for significantly small values of *ζ*, both *D* and *L*_*avg*_ tend to be small, since few nodes are connected. By then increasing *ζ*, *D* and *L*_*avg*_ also increase up to a maximum, connecting more nodes that had no previous connections, after which they tend to decrease, connecting nodes that were already connected. We find that the constructed networks tend to reach a stable point at or shortly after *D* and *L*_*avg*_ have reached a unique peak for uni-modal distributions, and at or shortly after the last of multiple peaks of *D* and *L*_*avg*_ for multi-modal distributions.

The selected value *ζ*_*s*_ carries much information. In general, a higher value suggests the distribution has a large spread and is therefore more heterogeneous, and a low value suggests the distribution is more homogeneous. We can therefore calculate a homogeneity information index by dividing *ζ*_*s*_ by the mode *M*: *H* = *ζ*_*s*_ */M*. For instance in Fig 1, the network becomes stable for *ζ* = 10, the highest degree is 39 for the node with value 98.5387, and therefore the homogeneity index of the distribution is *M* = 10/98.5387 = 0.1015. Although *H* is bounded by [0, ∞], it is most often within the range [0,1], since for values larger than 1, *ζ*_*s*_ > *M*, and all values below *M* are connected.

**Fig 2:**
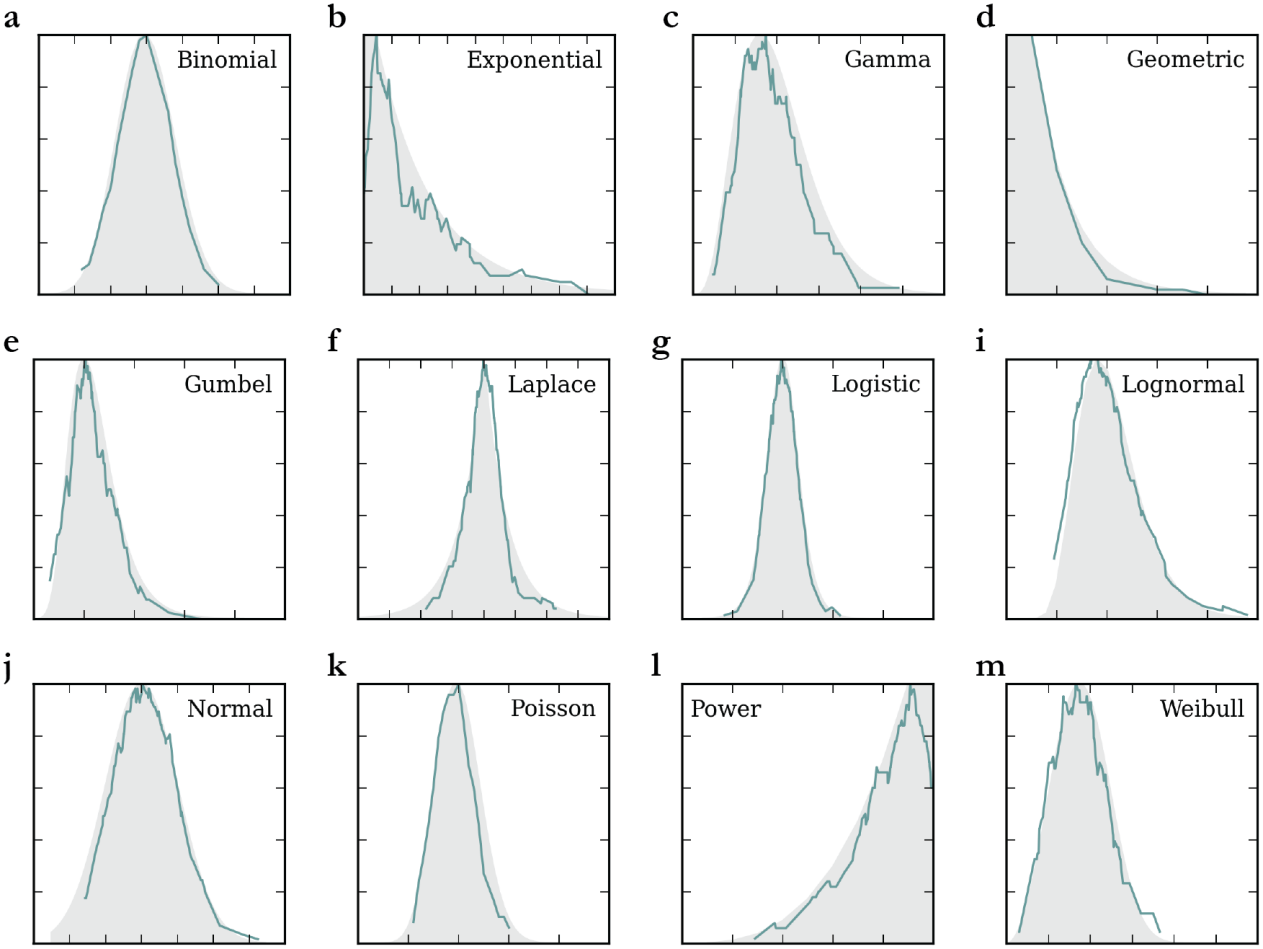
Application of NB Methodology on Twelve Simulated Distributions. For each type of distribution, 100 values were randomly simulated. The degree-value curves are shown in solid green lines, and the theoretical distributions are shown in shaded gray. The distributions and curves were standardized between 0 and 1 to fit on the same scale. The properties and results for each distribution are available in Section E in S1 File.

To insure the validity of the method, we then applied it to twelve traditional types of distributions, where we randomly sampled one hundred data points for each of them and where most of NB histograms follow closely the respective simulated distributions (Fig 2). Errors occur for distributions that do not possess a distinct mode such as the exponential and power distributions. By nature, the method links data points when they are close in value, which creates these artificial peaks in these cases. Despite this problem, these artificial peaks can be detected since they occur close to the minimum or maximum of a distribution (i.e., by the window boundaries). Compared to traditional histograms using Scott’s rule, our results perform as well or even better since the bin sizes selected are often too large (see Section E in S1 File).

### Application

**Fig 3:**
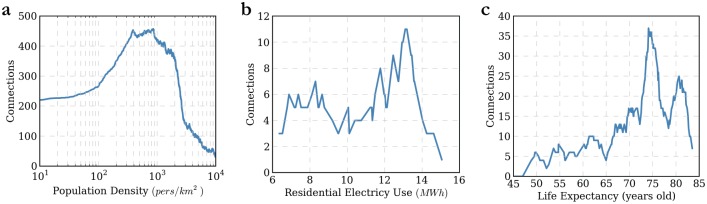
Application of Network-Based Methodology on Three Real-World Data Sets. (A) Chicago Metropolitan Statistical Area population density for 2,207 census tracts, where we used a log-scale because of the heterogeneity in the data (Source: 2010 US Census). (B) 2012 residential electricity use for the 50 US states and the District of Columbia (Source: EIA). (C) 2012 world life expectancy for 199 countries (Source: World Bank). See Section F in S1 File for details on each data set.

The NB histogram methodology can be applied to any data sets. To further illustrate its benefits, we apply it to three completely different data sets: (1) 2010 population density in Chicago, (2) 2012 residential electricity use in the United-States, (3) 2012 life expectancy in the world. The results for these three applications are shown in Fig 3 and tabulated in Table 2.

**Table 2:** Network-Based Methodology Application.

	*V*	*E*	*ζ*_*s*_	*M*	*H*	*p*_*g*_	*D*	*L*_*avg*_
**Chicago Population Density**	2,207	283,041	387.40	854	0.454	0.9887	46	9.28
**US Residential Electricity Use**	51	146	0.57	13.13	0.043	1.0000	21	7.28
**World Life Expectancy**	199	1,725	1.18	74.07	0.016	0.9899	33	9.74

The population density of an urban region is generally calculated by dividing the total population by the total surface area of the region. By taking the example of the Chicago Metropolitan Statistical Area, we get a value of 496 pers/km^2^. The problem is that by considering the total area, we essentially give a larger weight to large areas that have small number of residents, and this value of 496 pers/km^2^ is simply not representative of the region (see Section G in S1 File for a map and a cartogram). Another method is to consider population densities in individual census tracts, calculate the average, and locate the median. When doing this for Chicago, we get values of 3,546 pers/km^2^ and 2,039 pers/km^2^ respectively. Although these values are much more representative, they are heavily skewed to the right and they fail to capture the mode of the distribution. When applying the NB methodology to the 2,207 census tracts (Fig 3(A)), we find a mode of 854 pers/km^2^ with *ζ*_*s*_ = 387.4, which is both between the traditional value of population density and the mean, and it is arguably much more representative of the overall population density of the region. Moreover, the distribution is relatively heterogeneous with *H* = 0.45; the smallest value in the data set is 0 and the highest is 196,413 pers/km^2^. We also note that the method is also able to directly determine census tracts that vary greatly from the main trend simply because they are not in the giant cluster (and they are therefore outliers; see Outlier Detection section above).

In the US, electricity use per capita can vary greatly by state, with southern states as well as rural states generally consuming more electricity for space cooling, lighting and other household electrical needs. When looking at per household residential electricity use in the 50 US states and the District of Columbia, the average electricity use is 10.78 MWh and the median is 11.30 MWh. Again, these values are not representative of the overall trends. The average is essentially the average of the averages of all states, and thus low-populated states are therefore given heavier weights per household. When applying the NB method (Fig 3(B)), we can detect two general modes as opposed to one, with highest consumers tending to be southern states. The value of the right mode is 13.13, which is an average of two values (Georgia with 13.18 MWh and Nevada with 13.09 MWh; both with degrees of 11), and the value of the left mode is 8.30 MWh in New Jersey. Despite its bi-modal feature, the distribution is relatively homogenous with *H* = 0.04, ranging from a small value of 6.37 MWh in Maine to a maximum value of 15.04 MWh in Louisianna.

**Fig 4:**
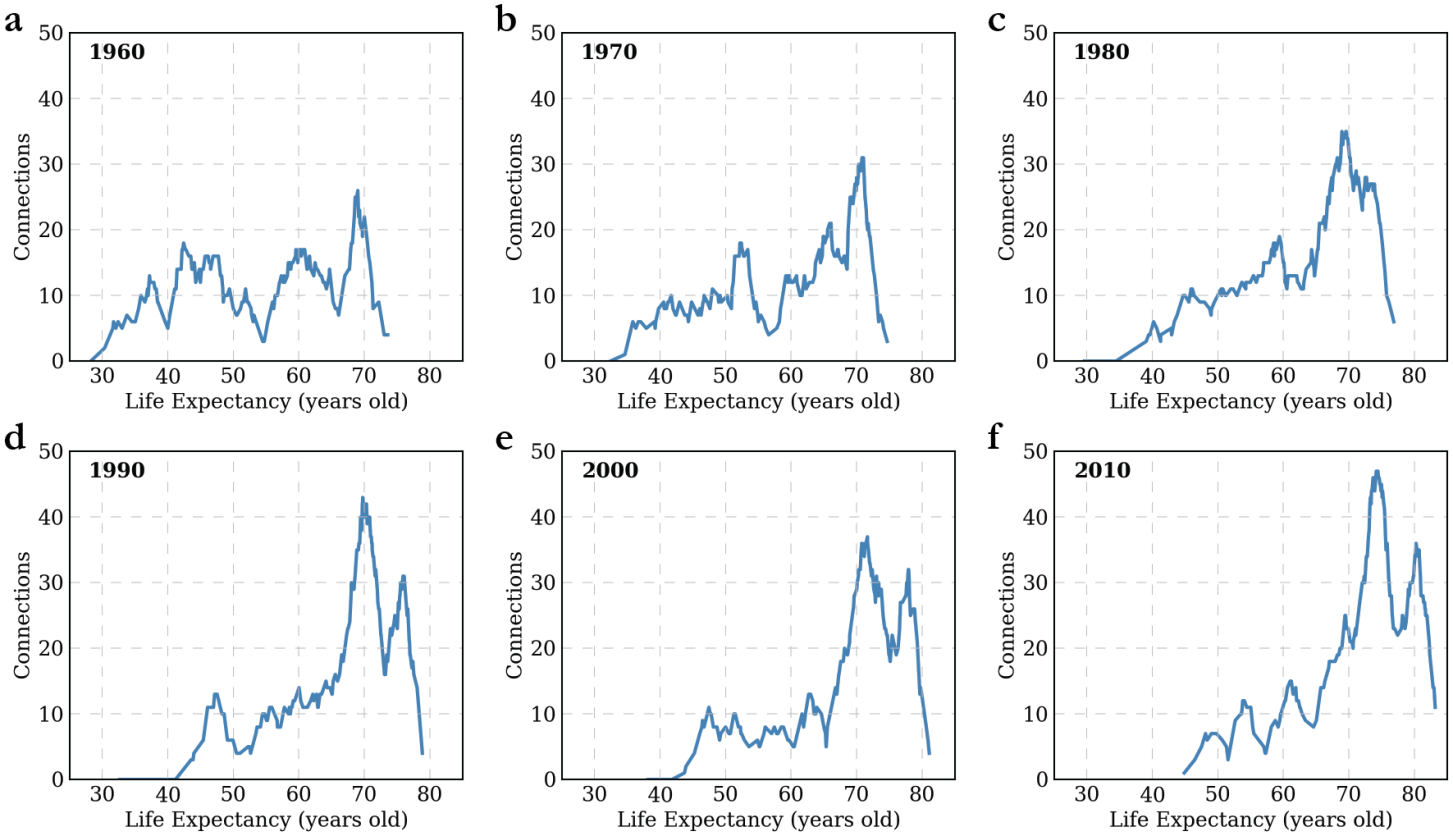
NB Histograms of Evolution of Life Expectancy in the World from 1960 to 2010. (A)-(F) 1960, 1970, 1980, 1990, 2000, 2010. (Data Source: World Bank)

Life expectancy is the main indicator of development for countries. The average life expectancy in the world in 2012 was 70.58 years old from the 199 countries available from the World Bank Data Catalogue. Again, this value does not take into account populations, and it is skewed by a significant number of countries that still strive to improve their life expectancy. The median is 73.5 years old. When applying the NB methodology (Fig 3(C)), we find a single mode with a value of 74.07 years old in Sri Lanka, although a small secondary mode also exists, right of the main mode that includes more developed countries. Fig 4(C) also highlights the significant proportion of countries with much lower life expectancies. The distribution is also homogenous with *H* = 0.016 with values ranging from 45.32 years old in Sierra Leone to 83.48 years old in Hong Kong.

Dwelling on life expectancy, we can also apply the NB methodology to longitudinal data sets and observe how overall patterns have evolved over time. Fig 4 shows that life expectancy has evolved significantly since 1960, from a distinct multi-modal distribution that does not show any significant patterns to the emergence of a main mode and a smaller secondary mode in 2010. See Section H in S1 File for details on each year.

## Conclusions

With the contemporary profusion of data, traditional statistical indicators are often insufficient to capture actual and meaningful trends in a phenomenon. To partially address this problem, the objective of this study was to introduce and develop a novel network-based and binless methodology to perform frequency analyses and plot histograms.

By comparing every single value of a data set with one another, the methodology links two values when they are similar to each other. Technically similar to the construction of a network, the node with the highest degree is assimilated to the mode of the distribution. Moreover, we use the proportional number of nodes in the largest cluster to determine *ζ*_*s*_. Thanks to its nature, outliers have little to no impact on the metrics calculated. This feature is particularly important since current data sets often have significant outliers that are hard to detect manually (e.g., from sensor data that generate millions of points). Moreover, the method can be applied to data sets of any size. In fact, because the range *ζ* is automatically adjusted, even small data sets with few points can be processed. On the other hand, because the method tests for multiple ranges as opposed to using one pre-determined bin size (as is the case for traditional techniques), the method is more computational-intensive.

Overall, the network-based methodology offers significant benefits that are much needed to gain accurate insights from the large number of data sets available nowadays. It also has much room for improvement, in particular by incorporating many metrics from the growing field of Network Science, which we hope can complement the current efforts described in this work.

## Supporting Information

S1 File Eight sections to explain some of the concepts introduced in this work and to further describe some of the results. (PDF)
