# Supplementary material for: Network-Based and Binless Frequency Analyses
Source: PLoS One. 2015 Nov 3;10(11):e0142108. doi: 10.1371/journal.pone.0142108 (PMC4631440; doi:10.1371/journal.pone.0142108)

## A. Mean vs. Mode to Represent a Distribution

Most distributions in real life are asymmetric, and the arithmetic mean of a distribution therefore rarely corresponds to the mode of the distribution. Despite this problem, the mean of a distribution is often used to describe overall trends and patterns in a system. Arguably, however, the mode of a distribution is much more representative of overall trends and should therefore be used to describe these trends.

For unimodal distribution (Fig. A1.a), this asymmetric property essentially skews the mean towards the side that has a larger tail, therefore missing the mode. For multimodal distributions (Fig. A1.b), the mean is located in-between the modes and therefore reveals false information about relevant trends.

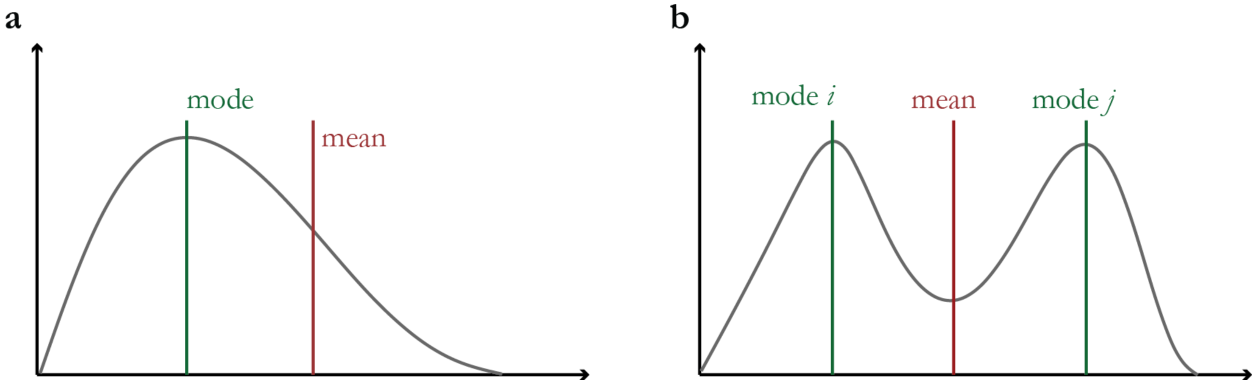


Figure A1 Mean and Mode. **a.** Uni-modal distributions. **b.** Multi-modal distributions

**B. Kernel Density Estimation**

Kernel density estimation (KDE) is a conventional machine learning technique to determine the probability density function of a dataset. Akin to the proposed methodology, it is non-parametric and it compares every single value of a dataset with one another within a certain threshold to construct a probability density function. KDE does not go beyond calculating a probability density function, however, unlike the proposed methodology.

The premise of KDE is to calculate a normal distribution for every data point taking its value as the mean. Various methods exist to assign a value for the standard deviation discussed briefly below. Mathematically, for a dataset of $N$ points, the probability $p(x)$ given any data point $x_i$ and a standard deviation $\sigma$ is:

$$p(x) = \frac{1}{N}\sum_{i=1}^{N}\mathcal{N}(x|x_i, \sigma^2)$$

where $\mathcal{N}()$ is the normal distribution. From [1], the approach can be generalized by:

$$p(x) = \frac{1}{N}\sum_{i=1}^{N}\kappa_h(x - x_i)$$

where $\kappa_h$ is the range set around each and every value, akin to the proposed methodology. This equation is also called a Parzen window density estimator.

One of the most common methods to determine $\kappa_h$ is the mean integrated squared error (MISE), which minimizes the squared error of the resulting distribution with the original data. Since there are no analytical solutions, this process can be relatively slow but comparable to the proposed methodology.

References

1. Murphy KP. Machine Learning: A Probabilistic Perspective. MIT Press; 2012.

### C. Simple and Illustrated Example for the Methodology

To illustrate the method, Fig. C1 shows a random sampling of 10 values from the normal distribution *N*(5,2). The left-hand side applies a traditional binning process, where we chose a bin width of 1 and the right-hand side applies the proposed method with $\zeta$ = 0.5, therefore representing a range of 1 as well. We can see that in this particular case, the NB histogram better captures the properties of the simulated distribution than the traditional histogram. Moreover, the traditional histogram places the mode of the distribution in a range [5,6), as opposed to a more desirable discrete value, compared to the NB methodology which gives 5.33 as the mode.

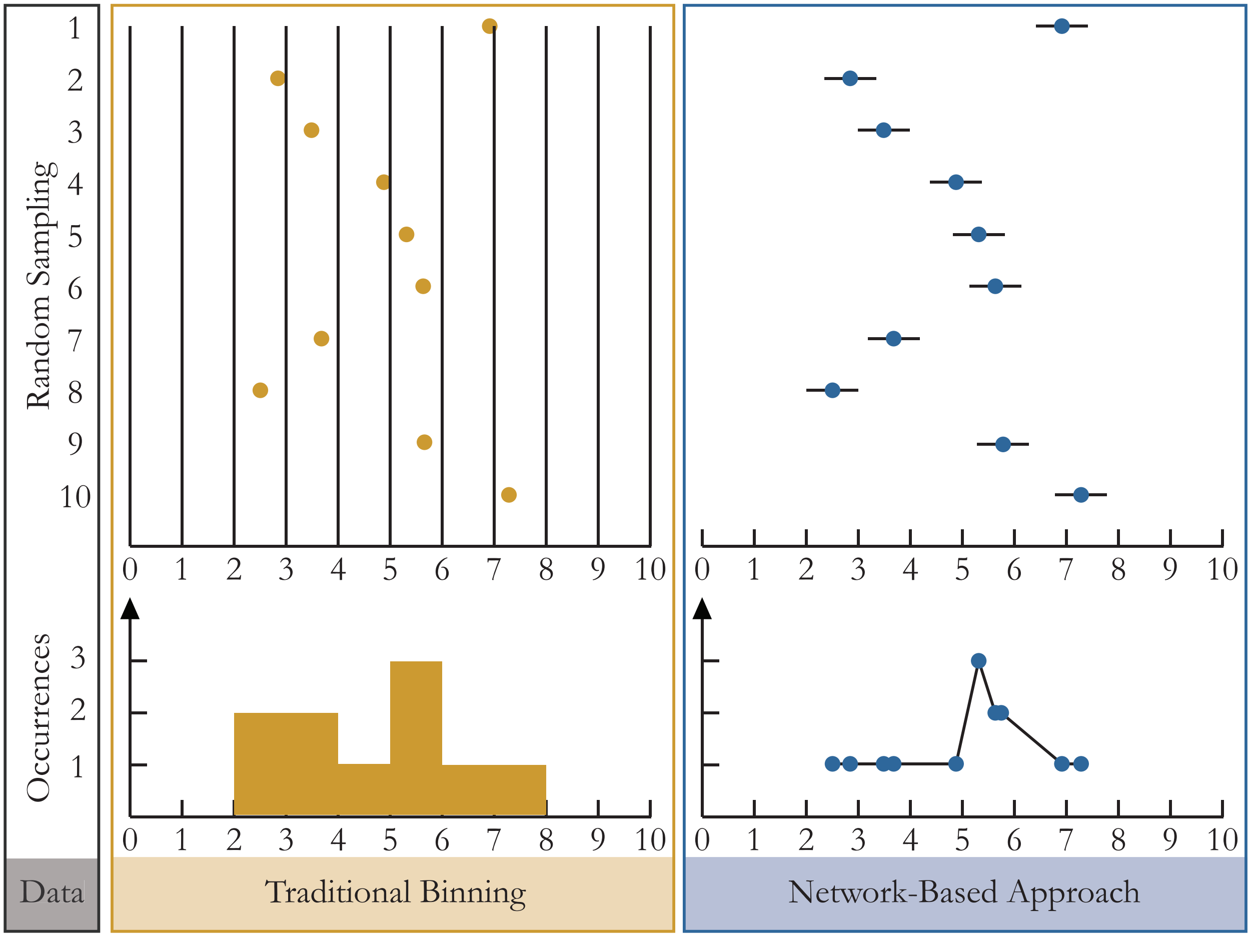


Figure C1 Illustration of the Network-Based (NB) Methodology. We randomly drew 10 values between 0 and 1 that fitted a normal distribution *N*(5,2); the 10 values are {6.90, 2.83, 3.49, 4.85, 5.33, 5.65, 3.69, 2.58, 5.74, 7.28}. Using a bin size of 1, the traditional binning approach on the left-hand side shows a peak of 3 occurrences in the interval [5,6). The network approach on the right-hand side shows a peak of degree 3 for the specific value 5.33 with a fixed $\zeta$ of 0.5.

**D. Python Script for Runtime Estimation**

```
#Libraries needed to run the code
import numpy as np
import pandas as pd
from igraph import *
import matplotlib.pyplot as plt
import datetime

#Open and copy data file
File_Name = raw_input("Name of file:")
File = pd.read_csv(File_Name + '.csv', header=None)

#Determine whether sampling is desired
sampling = raw_input("Data sampling, if yes, enter proportion (0 - 1) or leave blank:")

if sampling == "":
    distribution = np.array(File[0])
else:
    distribution = np.array(np.random.choice(File[0], int(float(sampling)*len(File.index)),
replace=False))

#Number of nodes
size = distribution.shape[0]

#Calculates median number to compile a list of zeta values to use
median = np.median(distribution)
zeta = np.arange(0.01*median, 0.11*median, 0.01*median)

#Create empty list to contain the results
networks = []

#Check start time
start = datetime.datetime.now()

#Main loop to create networks
for val in np.arange(10):
    adjacency = np.zeros(size**2).reshape(size, size)
    for i in np.arange(size):
        a =  distribution[i]-zeta[val] <= distribution
        b = distribution[i]+zeta[val] >= distribution
        adjacency[i] = a*b

    g = Graph.Adjacency((adjacency > 0).tolist(), mode=ADJ_UNDIRECTED)
    giant = g.clusters(WEAK).giant()
    networks.append([float(giant.vcount()) / g.vcount(), g, giant])
```

```
#Loop to determine the optimal network
step = 3
listTrack = []
giantSize = np.array([networks[x][0] for x in np.arange(len(networks))])
for i in np.arange(len(giantSize)):
    if i <= (len(giantSize) - step):
        track = (1/float(step))*sum(giantSize[i:i+step])/float(giantSize[i])
        listTrack.append(track)
    else:
        listTrack.append(1)

count = 0
running = 0
while running == 0:
    if 0.99999 < listTrack[count] < 1.00001:
        idNet = count
        running = 1
    else:
        count += 1

#Check end time and print time taken
end = datetime.datetime.now()
print("Time: {0}".format(end - start))

#Determine value of the mode and plot graph
list_max_degree = [networks[idNet][1].vs[idx].index for idx, li in
enumerate(networks[idNet][1].degree()) if li == np.max(networks[idNet][1].degree())]
index_max_degree = int(np.min(list_max_degree))
print("Optimal network id is {0} and mode value is {1}".format(idNet,
distribution[index_max_degree]))
plt.plot(distribution, networks[idNet][1].degree(), marker='o', linestyle="None")
plt.show()
plt.close()
```

**E. Method Validation And Distribution Properties**

The figures and information below show the twelve distributions that were selected for Fig. 2. We used the python library NumPy to randomly draw 100 points from each of these distributions. We then used the python library igraph to form the networks.

First, we list the equations for each distribution, the parameters selected, and the optimal cutoff value $\zeta_s$. Below the equations, we show the simulated network-based histogram in the solid green line and the theoretical distribution in the shaded gray. The figures were standardized between 0 and 1 so they could be superimposed.

Right of these figures, we overlay the theoretical distribution in shaded gray with a histogram in shaded blue that was produced using Scott's rule for bin sizing, where the bin size $b$ is defined as:

$$b = \frac{3.49\sigma}{n^{1/3}}$$

where $\sigma$ is the standard deviation and $n$ is the size of the population.

Below the main figures for each distribution, we show how the total number of edges/links $E$, the proportional number of nodes in the largest cluster $p_g$, and the diameter $D$ and average path length $L_{avg}$ respectively evolve as a function of $\zeta$.

To determine $\zeta_s$, we first picked the median of the distribution and assigned $\zeta$ as 1% of the median, followed by 2% of the median, and so on and so forth until 50% of the median. For each resulting network, we measured $p_g = V_g / V$. The cutoff $\zeta_s$ was selected when $p_g$ remained identical for eight consecutive increase in the cutoff $\zeta$. Eight was selected as a fairly standard statistical value, but it can be increased or decreased depending on the selection of intervals between each $\zeta$.

Binomial Distribution

$$P(x) = \frac{N!}{x!(N-x)!} p^x (1-p)^{N-x}$$

$N = 100$
$p = 0.2$
$\zeta_s = 2$

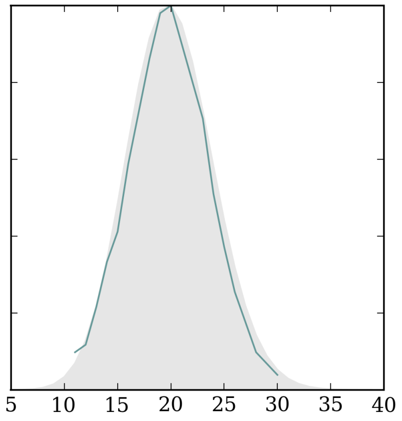


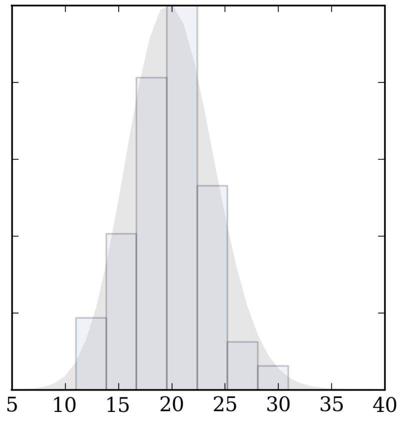


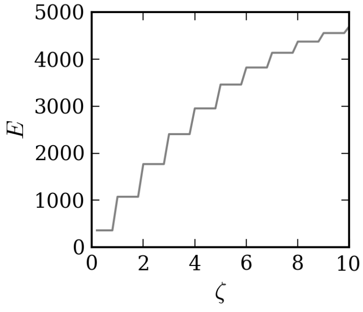


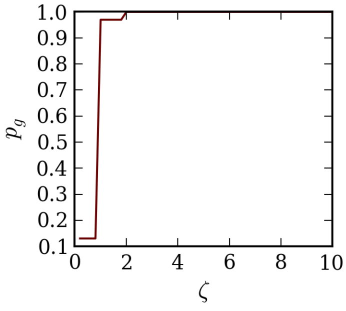


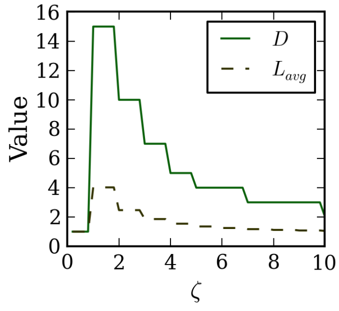

Exponential Distribution

$$f(x, \frac{1}{\beta}) = \frac{1}{\beta} exp(-\frac{x}{\beta})$$
$$\beta = 10$$
$$\zeta_s = 2.3$$

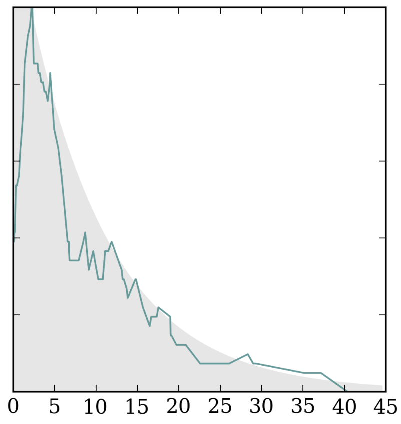


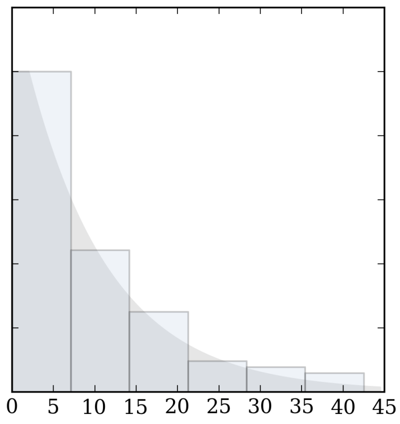


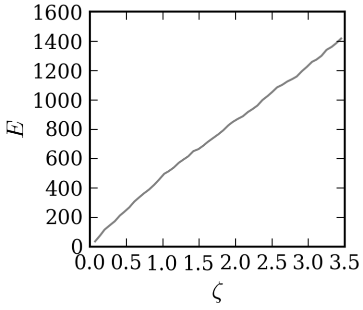


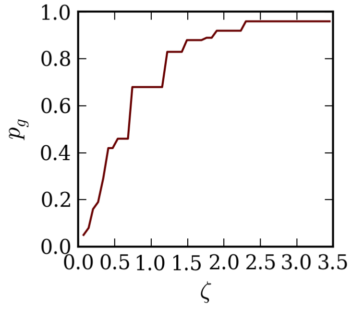


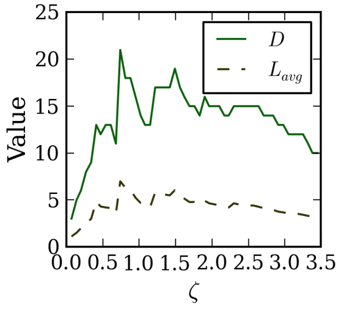

## Gamma Distribution

$$p(x) = x^{k-1} \frac{e^{-x/\theta}}{\theta^k \Gamma(k)}$$

$k = 5$
$\theta = 2$
$\zeta_s = 1.74$

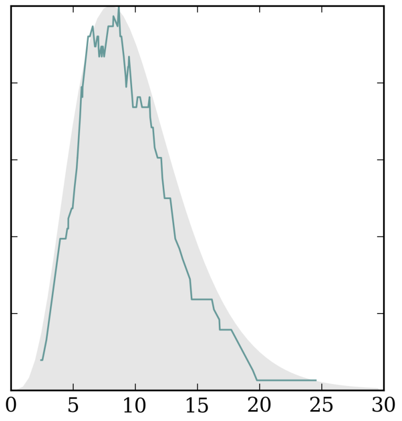


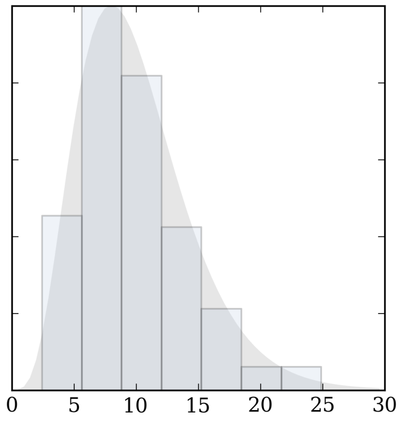


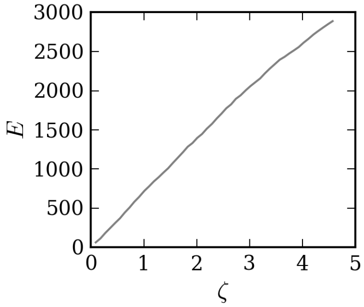


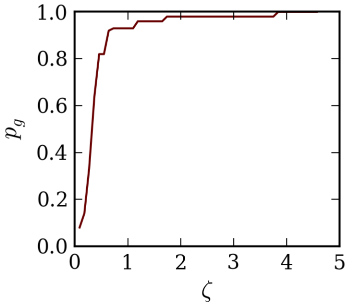


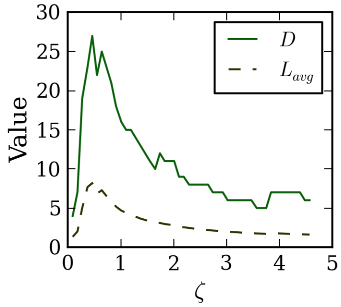

Geometric Distribution

$f(k) = (1-p)^{k-1}p$
$p = 0.5$
$\zeta_s = 0.01$

Comment: all numbers generated are necessarily integers, which is why the network properties do not evolve by increasing $\zeta$.

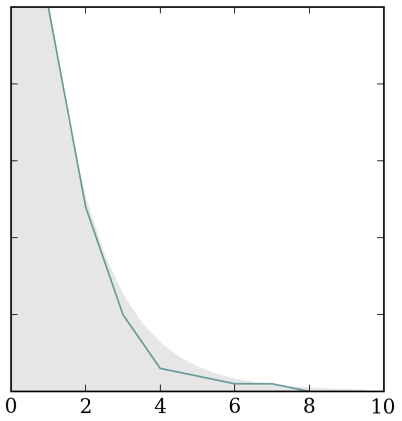


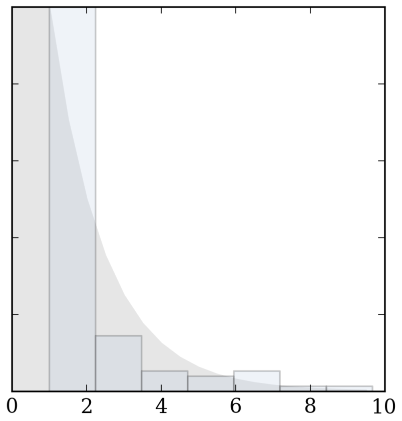


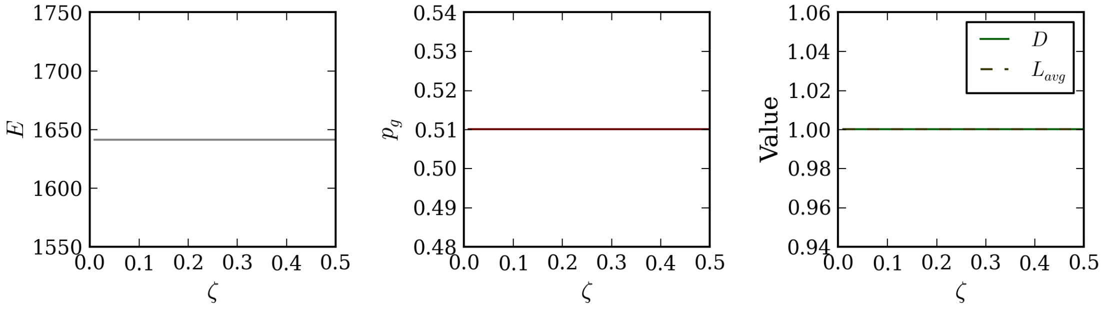

## Gumbel Distribution

$$p(x) = \frac{-(x-\mu)/\beta}{\beta} e^{e^{-(x-\mu)/\beta}}$$

$\mu = 100$

$\beta = 20$

$\zeta_s = 10.51$

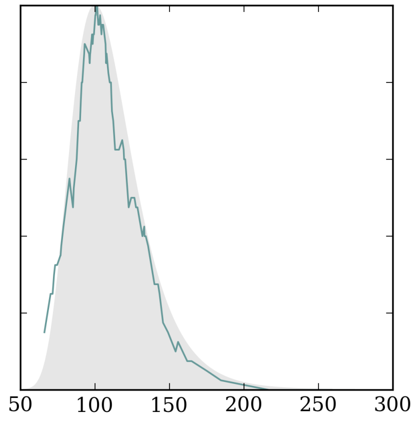


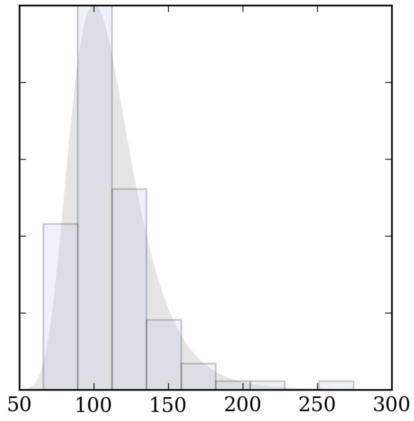


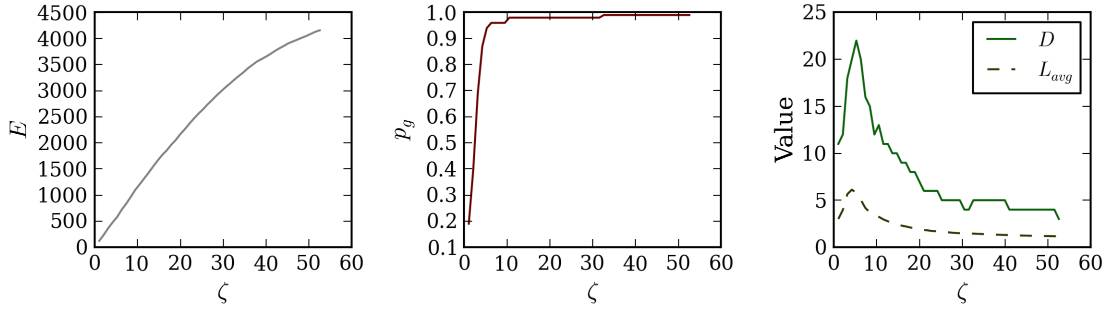

Laplace Distribution

$$f(x;\mu,\lambda) = \frac{1}{2\lambda} exp(-\frac{|x-\mu|}{\lambda})$$

$\mu = 100$

$\lambda = 15$

$\zeta_s = 7.04$

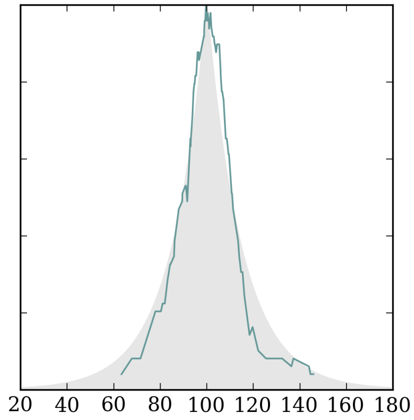


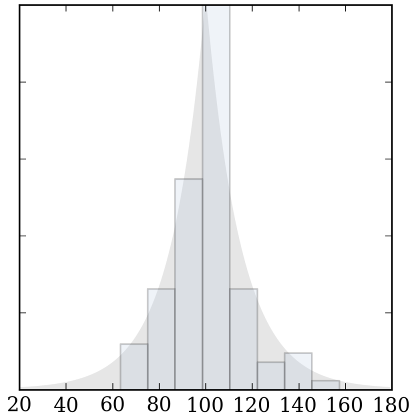


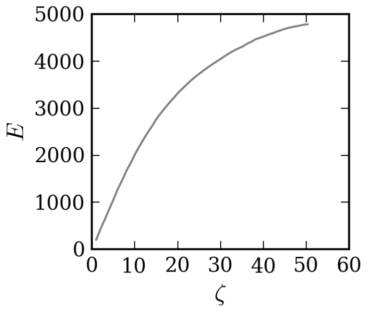


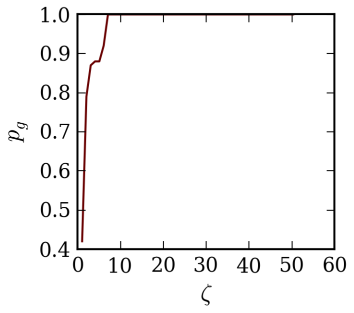


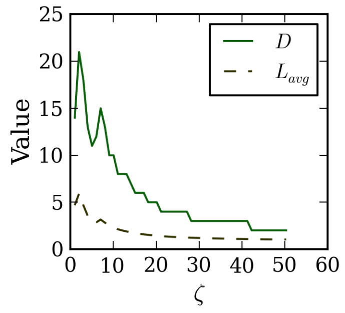

## Logistic Distribution

$$P(x) = \frac{e^{-(x-\mu)/s}}{s(1+e^{-(x-\mu)/s})^2}$$

$\mu = 100$

$s = 10$

$\zeta_s = 12.69$

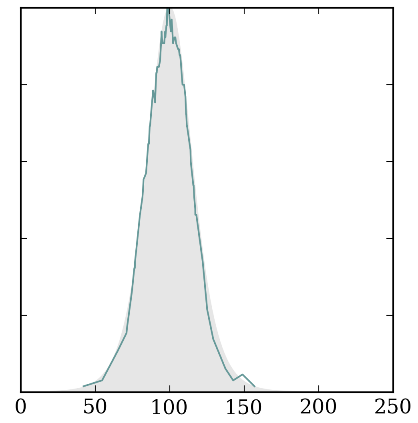


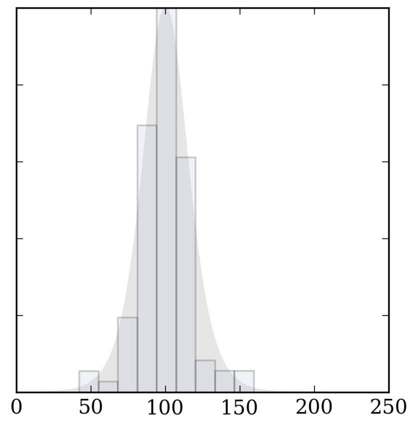


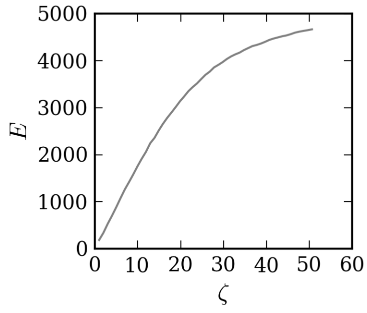


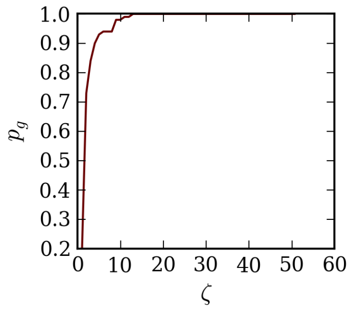


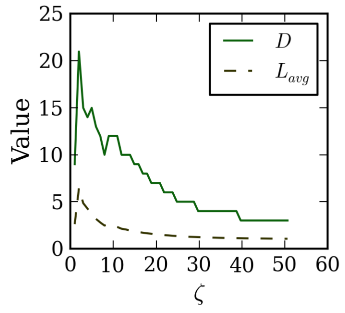

Lognormal Distribution

$$p(x) = \frac{1}{\sigma x \sqrt{2\pi}} exp^{-\frac{(ln(x)-\mu)^2}{2\sigma^2}}$$

$\mu = 0$

$\sigma = 0.3$

$\zeta_s = 0.24$

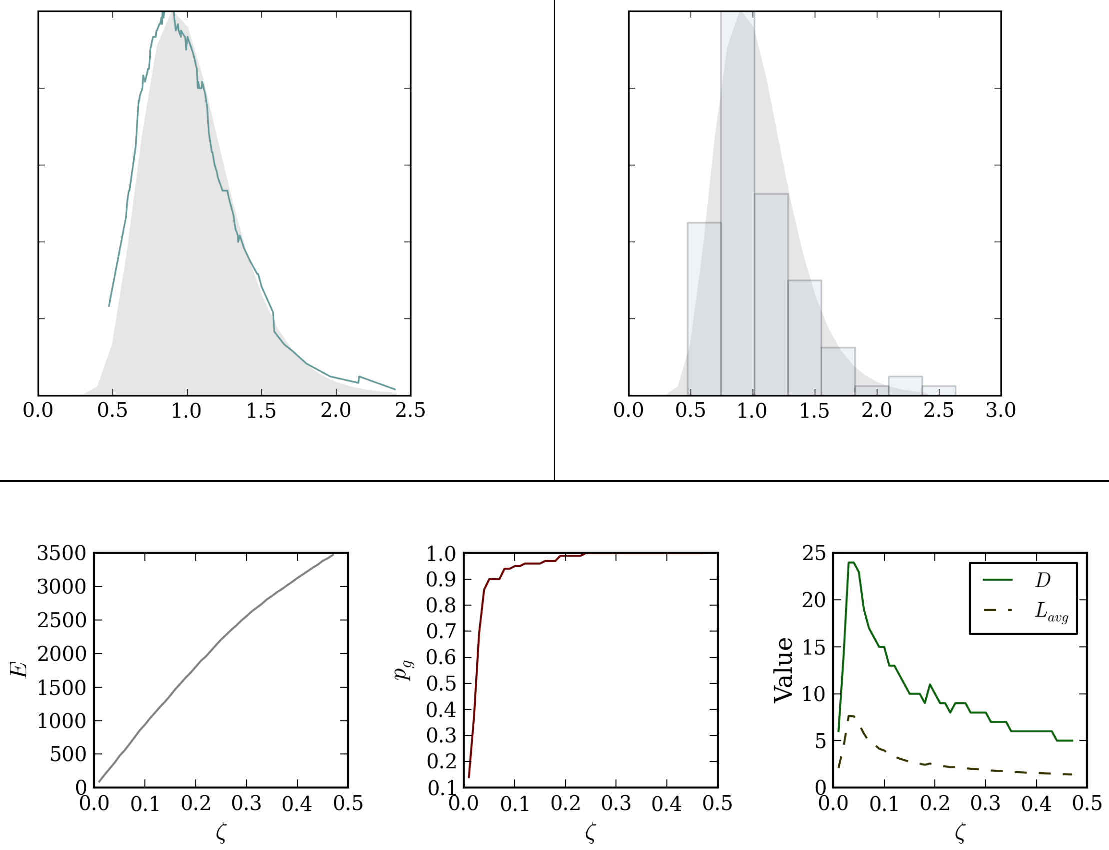

## Normal Distribution

$$p(x) = \frac{1}{\sqrt{2\pi\sigma^2}} e^{-\frac{(x-\mu)^2}{2\sigma^2}}$$

$\mu = 100$

$\sigma = 20$

$\zeta_s = 9.96$

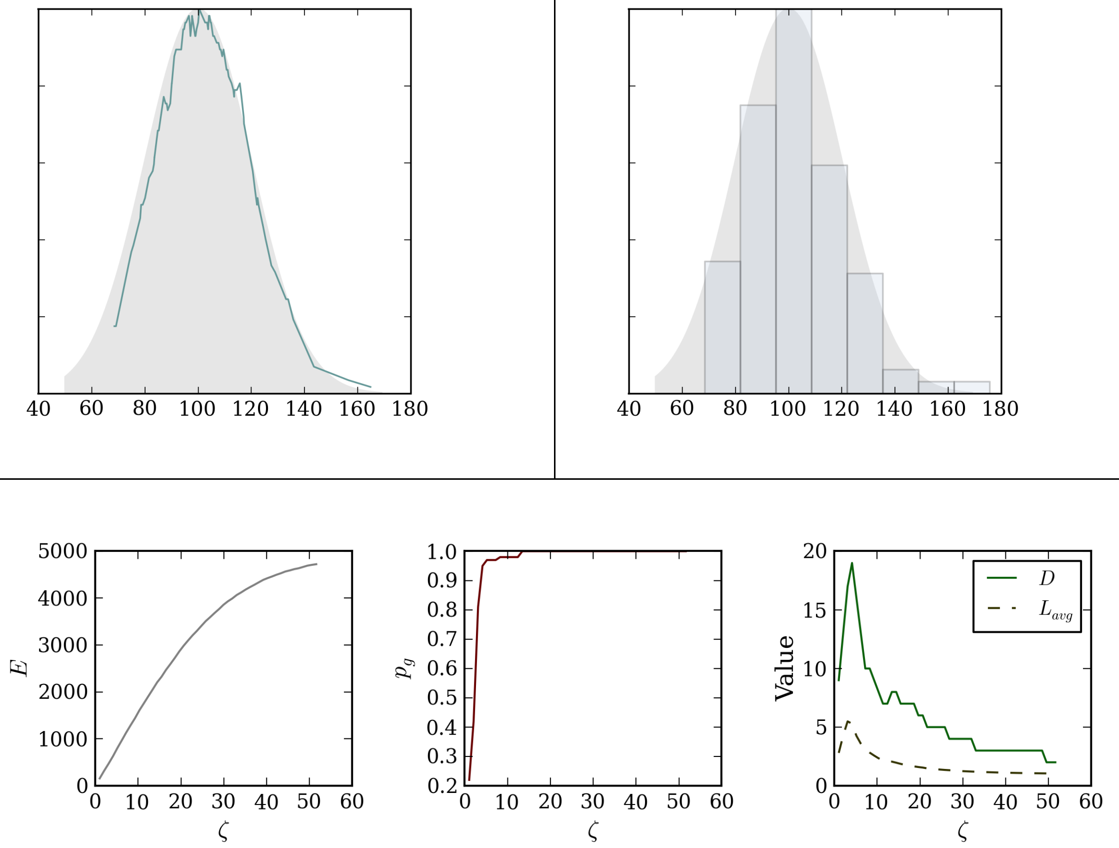

Poisson Distribution

$$f(k;\lambda) = \frac{\lambda^k e^{-\lambda}}{k!}$$

$\lambda = 20$
$\zeta_s = 2.09$

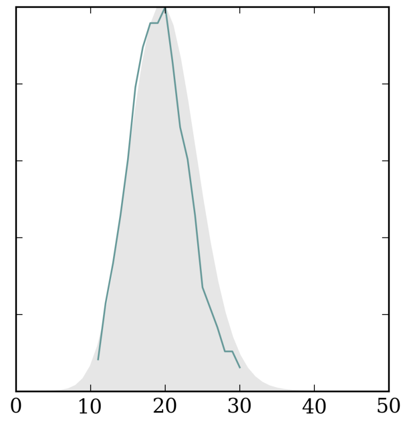


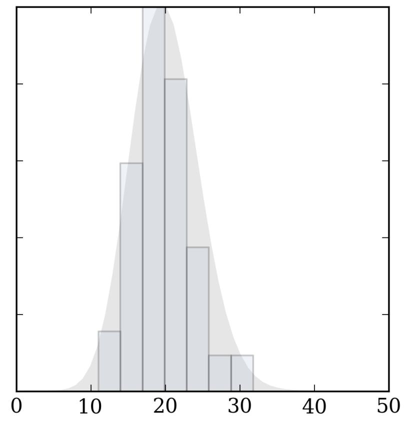


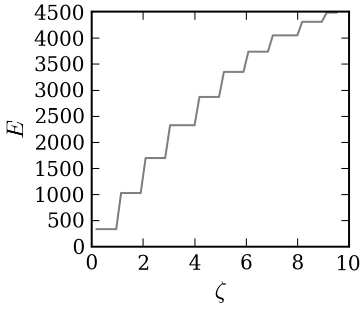


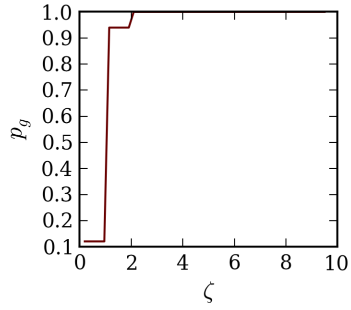


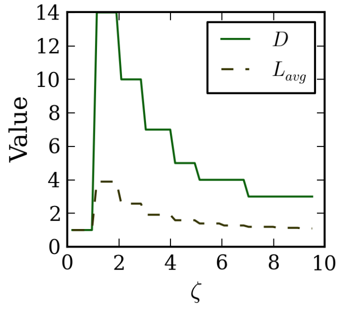

Power Distribution

$$P(x;a) = ax^{a-1}, 0 \leq x \leq 1, a > 0$$

$a = 4$

$\zeta_s = 0.08$

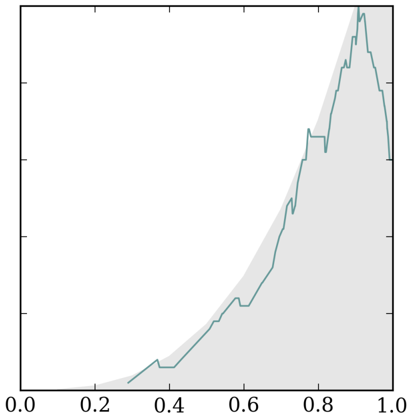


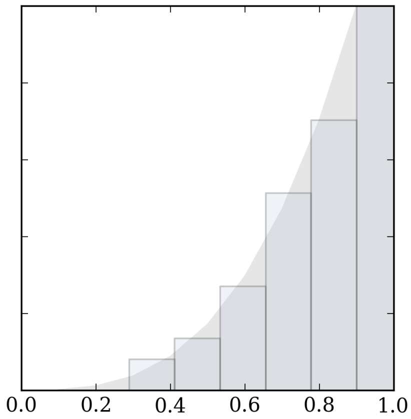


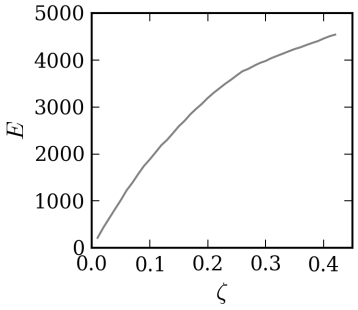


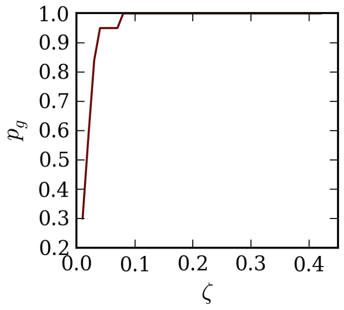


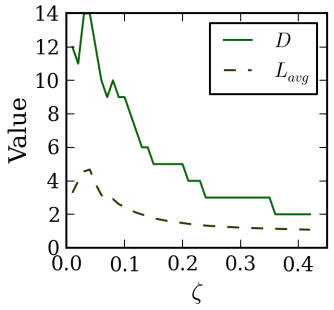

Weibull Distribution

$$p(x;a,\lambda) = \frac{a}{\lambda}(\frac{x}{\lambda})^{a-1}e^{-(x/\lambda)^a}$$

$a = 3$
$\lambda = 1$
$\zeta_s = 0.19$

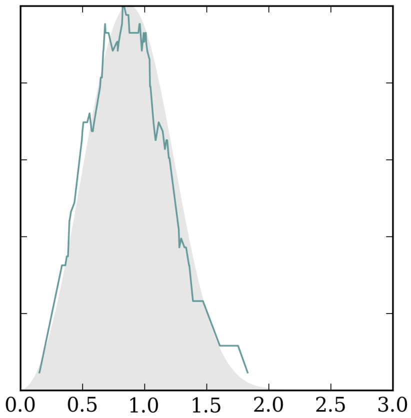


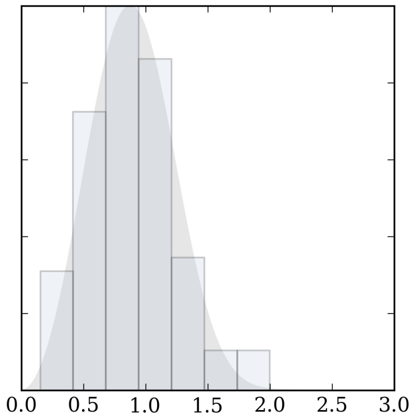


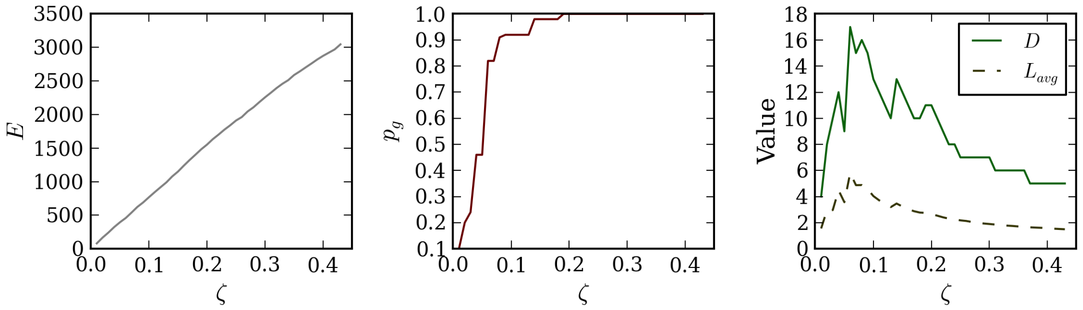

**F. Network Properties of Three Real-World Applications**

A similar procedure as the one described in S1 E was employed for the three real-world applications. For life, however, the increment was modified from 1% of the median to 0.1% of the median considering the large number of points (i.e., 199 countries) and the relatively small scale of possible values (from 45.32 to 83.48 years old).

The figures below show first the histogram that was produced using Scott's rule for bin sizing, and then the graph properties of the selected networks in the following order: number of links, evolution of $p_g$, and evolution of $D$ and $L_{avg}$.

2010 Chicago Population Density

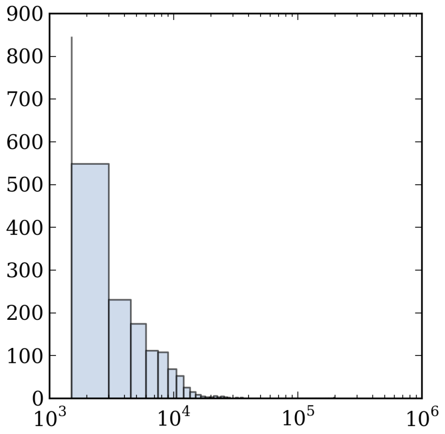


Comment: the x-axis is on a log scale. Because of the heterogeneity of the data, the initial mode is concealed on the histogram

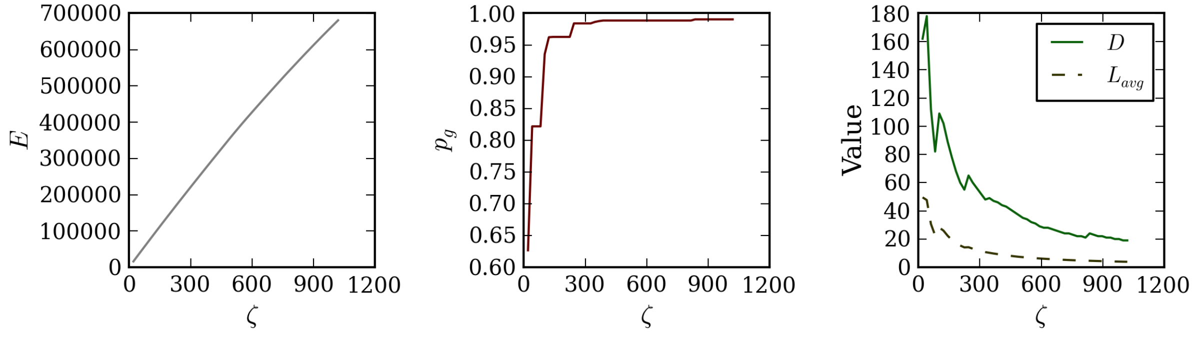

## 2012 US Residential Electricity

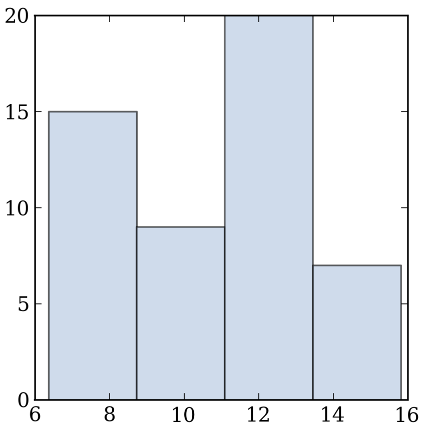


Comment: although the histogram captures the bi-modal property of the distribution, the size of the bins is too large to reveal any significant information.

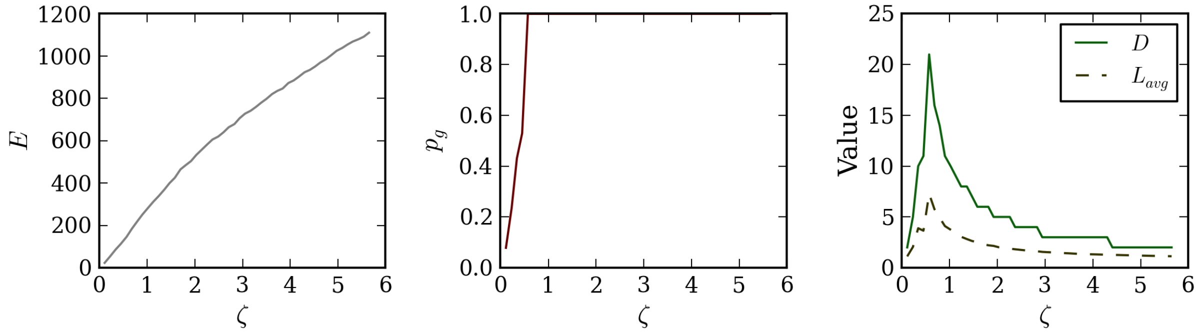

2012 World Life Expectancy

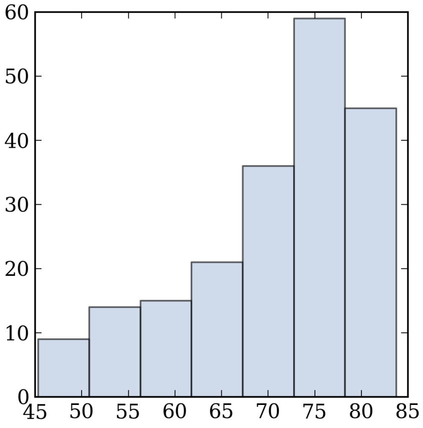


Comment: here again, the calculation of bin size is too large to reveal any significant information, and the presence of a second mode, right of the main mode, is concealed.

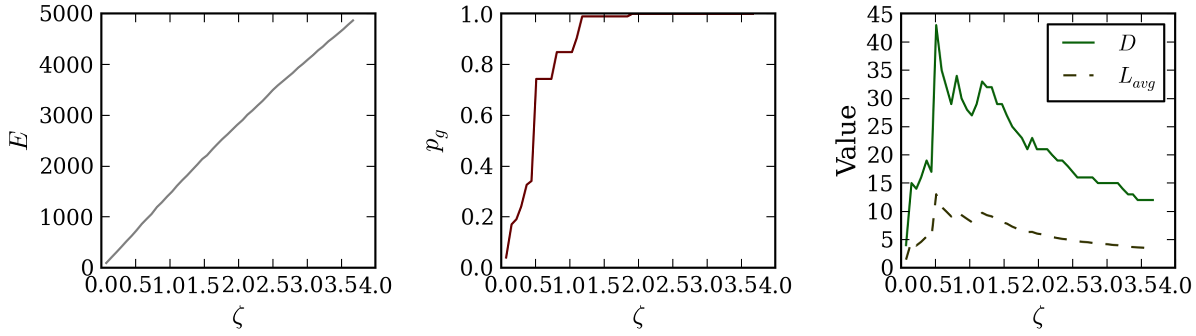

## G. Population Density in the Chicago Metropolitan Statistical Area (MSA)

Figure G1 shows a map of the 2,207 census tracts in the Chicago MSA. The categories of colors were carefully selected to highlight the fact that the traditionally reported population density of 496 pers/km$^2$ does not represent well the region. We can clearly see from this map that peripheral census tracts with low population densities have large areas that heavily impact the measurement of population density.

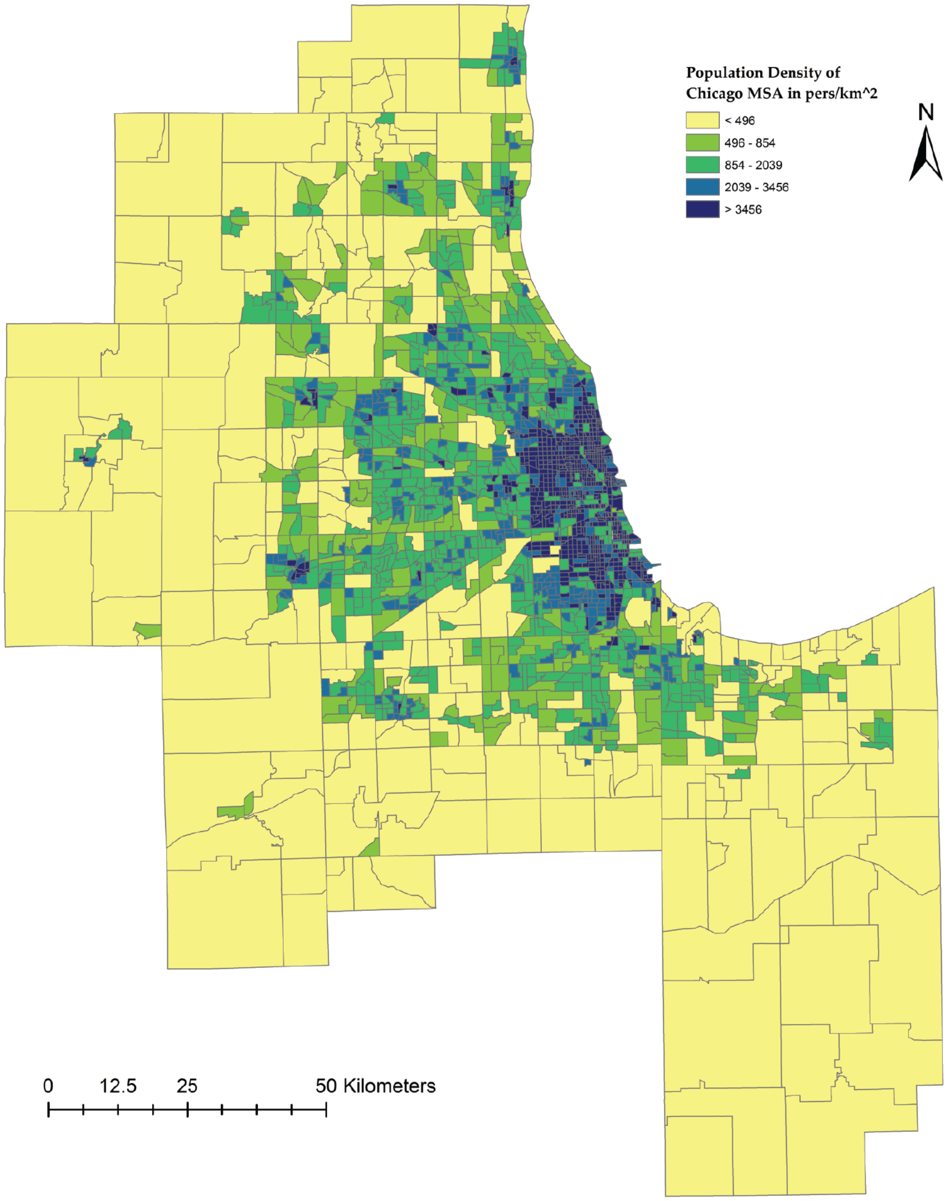


Figure G1 Map of Population Density in the Chicago Metropolitan Statistical Area (MSA). The census tracts are divided in 5 categories based on the measured population density, the calculated mode, the median and the average population density. The map was produced using ArcGIS 10.2.1.

In contrast, Figure G2 shows a cartogram of the Chicago MSA where the polygons of the census tracts have been redrawn and weighted by population. We can now clearly see that the large peripheral census tracts do not house a large proportion of the population. In contrast, the number of census tracts around the calculated mode is significant. By definition, half the census tracts have a population density higher than the median (i.e., the last two categories), attesting the heavily skewed feature of population density (i.e., large right tail), hence the use of a log-scale in Fig. 3.

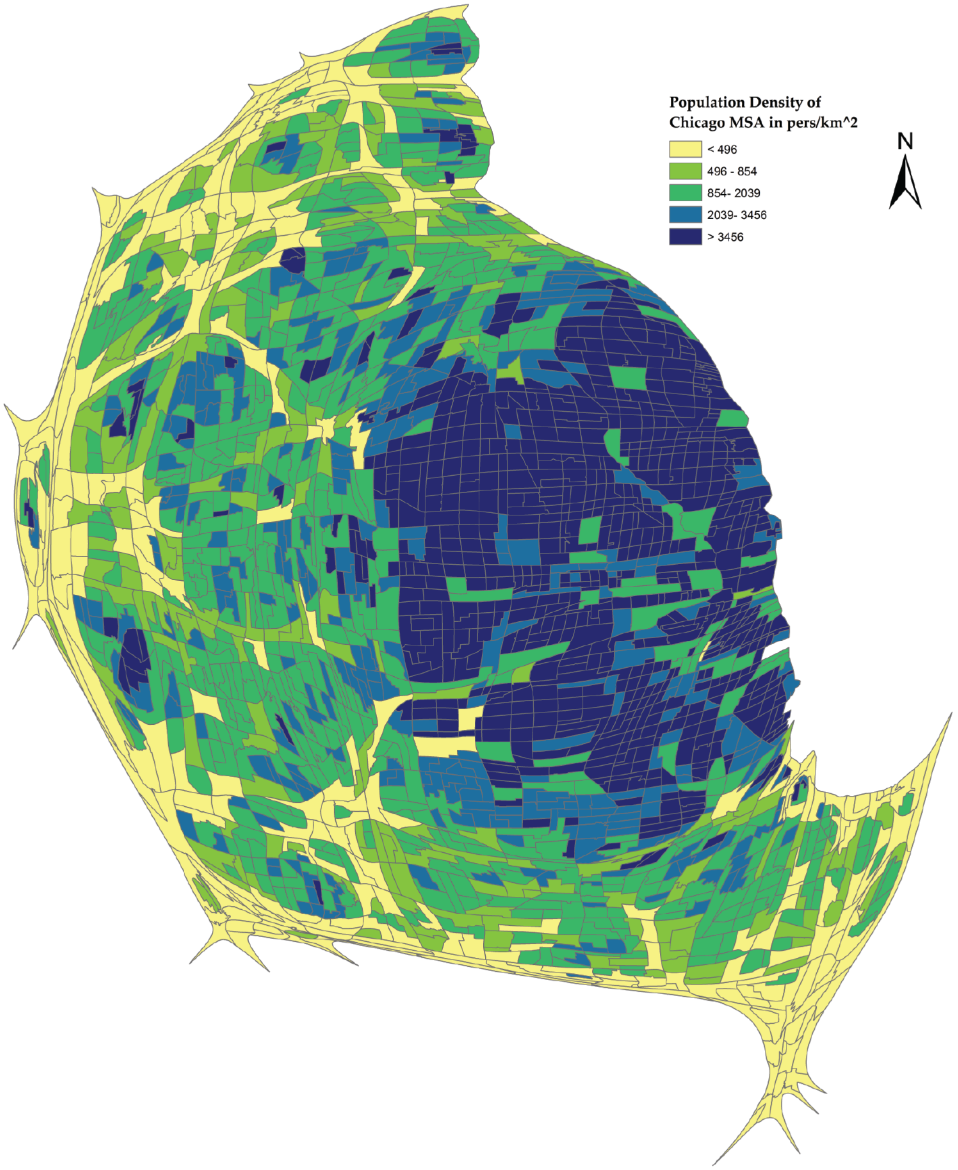


Figure G2 Cartogram of Population Density in the Chicago Metropolitan Statistical Area (MSA). The size of the census tracts is weighted based on their population. The census tracts are also divided in 5 categories based on the measured population density, the calculated mode, the median and the average population density. The map was produced using ArcGIS 10.2.1 and the ArcGIS tool to produce the cartogram is Cartogram Geoprocessing Tool 2.

### H. Longitudinal Study of Life Expectancy

Unlike the other applications, we started with a $\zeta = 0.1$, since life expectancy ranged from 28.21 years old (Mali in 1960) to 83.16 years old (San Marino in 2010). We then increased $\zeta$ by 0.1 years until 3.5. Moreover, a threshold of 0.68 was set for $p_g$, and we reduced the number of consecutive identical $p_g$ values to 6 because of this threshold. The eventual selection of $\zeta$ tends to be around the last peak in diameter.

The figures below show first the histogram that was produced using Scott's rule for bin sizing, and then the graph properties of the selected networks in the following order: number of links, evolution of $p_g$, and evolution of $D$ and $L_{avg}$. Here again we can see that the traditional histograms fail to capture relevant information.

**Life Expectancy 1960**

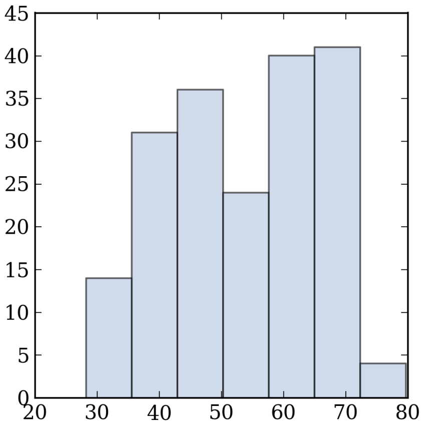


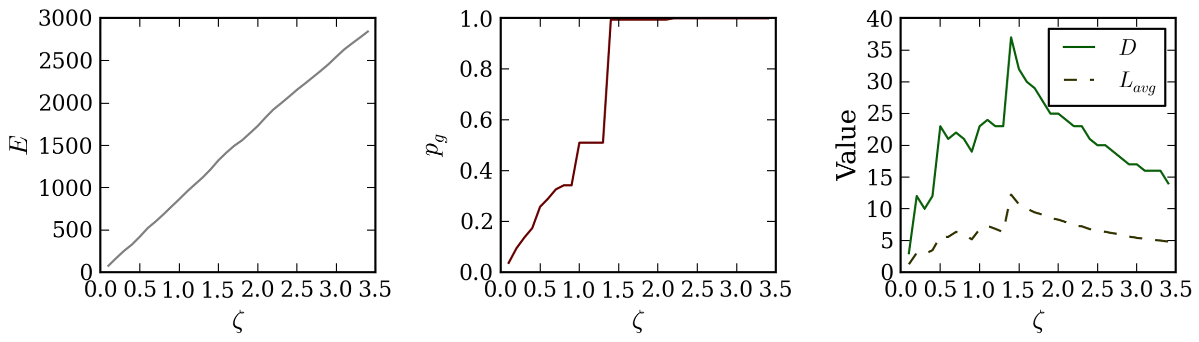

**Life Expectancy 1970**

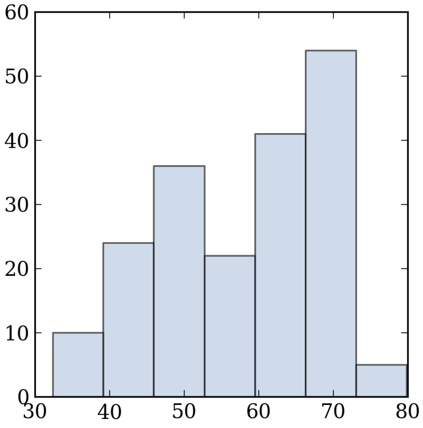


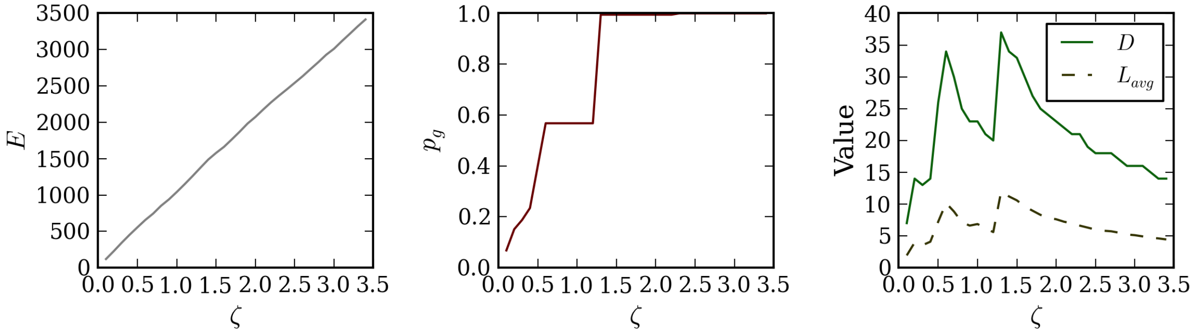

**Life Expectancy 1980**

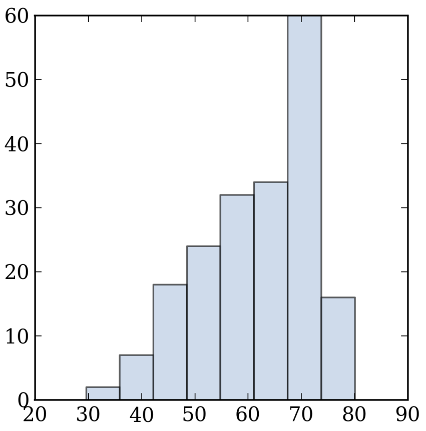


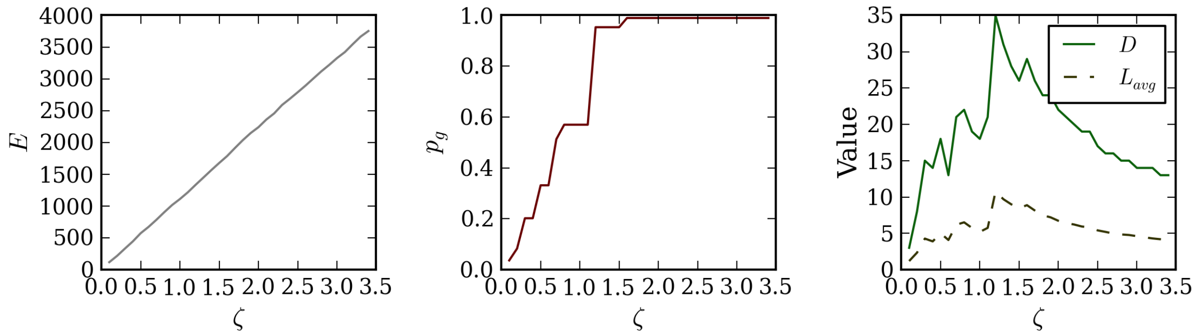

**Life Expectancy 1990**

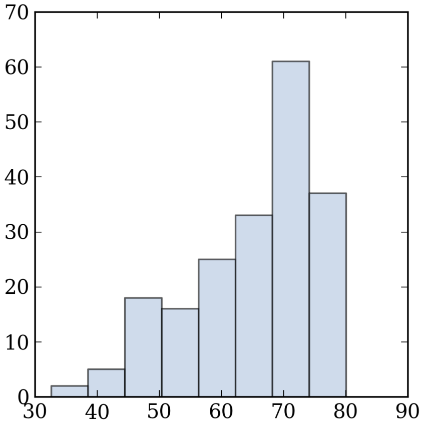

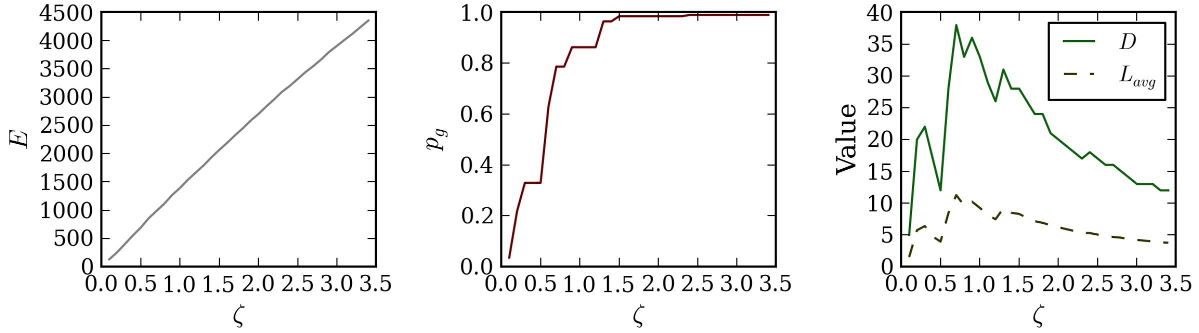

**Life Expectancy 2000**

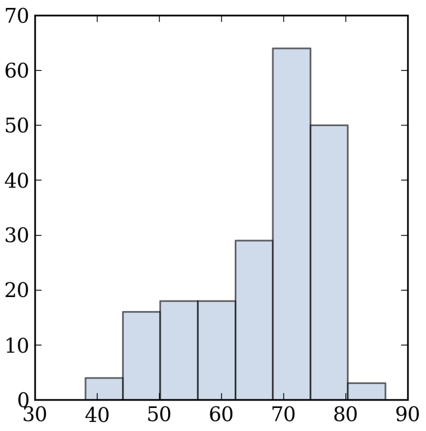

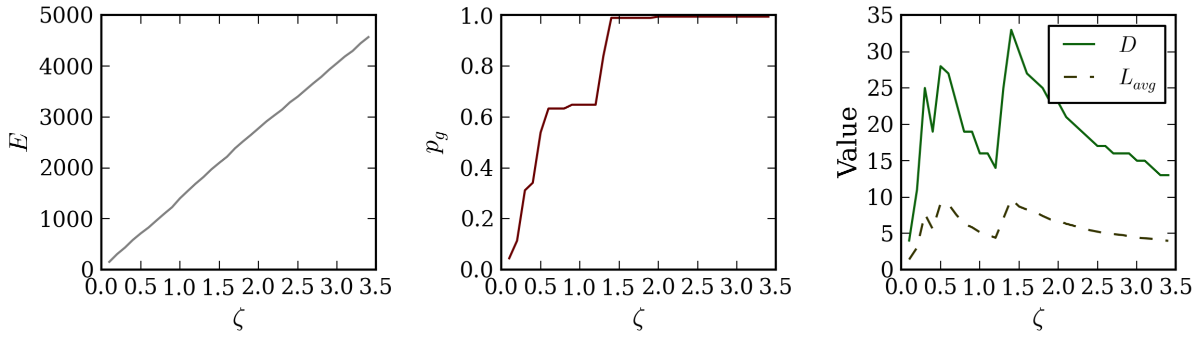

**Life Expectancy 2010**

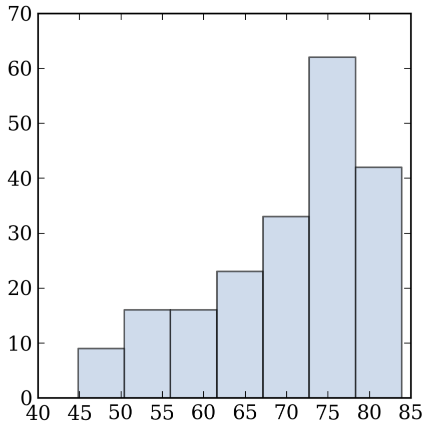


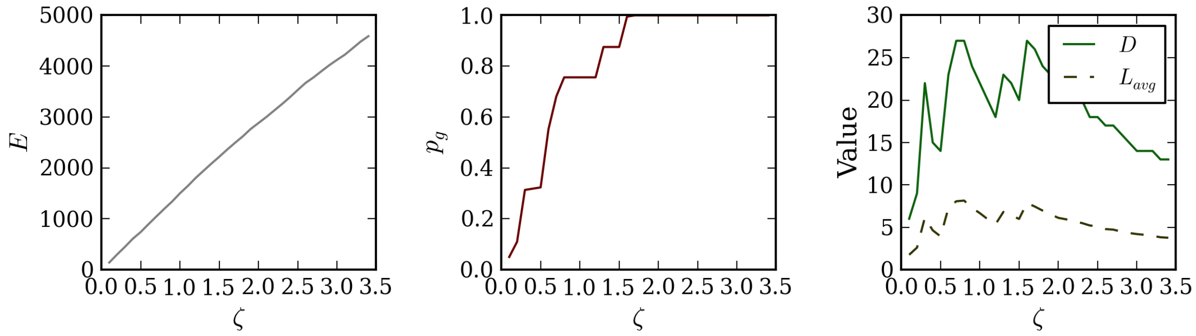

Supplement: S1 File — (PDF) [file pone.0142108.s001.pdf]
